# Implementation of an Electronic Patient-Reported Outcome App for Health-Related Quality of Life in Breast Cancer Patients: Evaluation and Acceptability Analysis in a Two-Center Prospective Trial

**DOI:** 10.2196/16128

**Published:** 2022-02-08

**Authors:** Joachim Graf, Nina Sickenberger, Katharina Brusniak, Lina Maria Matthies, Thomas M Deutsch, Elisabeth Simoes, Claudia Plappert, Lucia Keilmann, Andreas Hartkopf, Christina Barbara Walter, Markus Hahn, Tobias Engler, Stephanie Wallwiener, Florian Schuetz, Peter A Fasching, Andreas Schneeweiss, Sara Yvonne Brucker, Markus Wallwiener

**Affiliations:** 1 Institute for Health Sciences, Section of Midwifery Science University Hospital Tübingen Tübingen Germany; 2 Hospital for General Obstetrics and Gynecology University Hospital Heidelberg Heidelberg Germany; 3 National Center for Tumor Diseases University Hospital and German Cancer Research Center Heidelberg Germany; 4 Department of Women’s Health University Hospital Tübingen Tübingen Germany; 5 Department of Women’s Health Research Institute for Women’s Health University Hospital Tübingen Tübingen Germany; 6 Department of Obstetrics and Gynecology University Hospital Ludwig-Maximilians-University Munich München Germany; 7 Diakonissen-Stiftungs-Krankenhaus Speyer Speyer Germany; 8 Department of Gynecology and Obstetrics University Hospital Erlangen Erlangen Germany; 9 University Breast Center Franconia, Comprehensive Cancer Center Erlangen-EMN, Friedrich-Alexander University Erlangen-Nuremberg Erlangen Germany

**Keywords:** eHealth, electronic patient-reported outcomes, evaluation, acceptability, breast cancer

## Abstract

**Background:**

One in eight women is diagnosed with breast cancer in the course of their life. As systematic palliative treatment has only a limited effect on survival rates, the concept of health-related quality of life (HRQoL) was developed for measurement of patient-centered outcomes. Various studies have already demonstrated the reliability of paper-based patient-reported outcome (pPRO) and electronic patient-reported outcome (ePRO) surveys and that the 2 means of assessment are equally valid.

**Objective:**

The aim of this study was to analyze the acceptance and evaluation of a tablet-based ePRO app for breast cancer patients and to examine its suitability, effort, and difficulty in the context of HRQoL and sociodemographic factors.

**Methods:**

Overall, 106 women with adjuvant or advanced breast cancer were included in a 2-center study at 2 major university hospitals in Germany. Patients were asked to answer HRQoL and PRO questionnaires both on a tablet on-site using a specific eHealth assessment website and on paper. The suitability, effort, and difficulty of the app and self-reported technical skills were also assessed. Only the results of the electronically acquired data are presented here. The results of the reliability of the pPRO data have already been published elsewhere.

**Results:**

Patients regarded the ePRO assessment as more suitable (80/106, 75.5%), less stressful (73/106, 68.9%), and less difficult (69/106, 65.1%) than pPRO. The majority of patients stated that ePRO assessment improves health care in hospitals (87/106, 82.1%). However, evaluation of ePROs depended on the level of education (*P*=.003) in the dimensions of effort and difficulty (regression analysis). The app was rated highly in all categories. HRQoL data and therapy setting did not show significant correlations with the app’s evaluation parameters.

**Conclusions:**

The results indicate that ePRO surveys are feasible for measuring HRQoL in breast cancer patients and that those patients prefer ePRO assessment to pPRO assessment. It can also be seen that patients consider ePRO assessment to improve hospital health care. However, studies with larger numbers of patients are needed to develop apps that address the needs of patients with lower levels of education and technical skills.

## Introduction

### Breast Cancer: Epidemiological Relevance

With about 70,000 new cases each year, breast cancer is the most common cancer in Germany. One in eight women is diagnosed with breast cancer in the course of their life [1]. Due to great advances in cancer therapy options, the relative 5-year survival rate after initial diagnosis has increased to 88% [2]. While patients in adjuvant situations have an improved prognosis, patients with metastatic disease remain incurable and are hence treated with palliative care. Since currently systemic palliative treatment has a limited effect on survival rates, the concept of health-related quality of life (HRQoL) and measurement of patient-reported outcomes (PROs) are gaining increasing importance in the therapy of progressive diseases, such as breast cancer, especially in the metastatic setting [3-7]. Under this directive, the issue of how HRQoL data can be collected as efficiently and accurately as possible in real-world settings is gaining importance [8].

### PROs as a Holistic Addition to Clinic-Reported Outcomes

Drug evaluation studies have focused on clinical endpoints (clinic-reported outcomes), such as overall survival and progression-free survival, for years. Yet, PROs are becoming increasingly important to verify and compare the efficacy of different chemotherapeutic interventions in drug evaluation studies, not least owing to legal regulations [9]. This fact is confirmed by the enormous increase in studies publishing PRO data over the last few decades [10]. A PRO is widely defined as “any report of the status of a patient’s health condition that comes directly from the patient, without interpretation of the patient’s response by a clinician or anyone else” [11]. The concept of PRO takes into account the patient’s point of view concerning health status, therapy intervention, negative side effects, mental and functional components, satisfaction with care, drug adherence, and impact of progressive disease [9-14].

### Potential of Electronic PROs

Strong willingness to use technology within the population and existing infrastructure represents the rationale for digitalization in many sectors, including health [15]. While PROs are still routinely captured via paper-based methods, technical progress is gradually allowing more PRO data to be collected in the form of electronic PROs (ePROs), that is, via tablet computers or smartphones [16]. To ensure that patients are able to deal with ePRO data capture, studies that prove reliability and acceptance are needed. Various studies have already demonstrated that both paper-based PRO (pPRO) and ePRO surveys are reliable and equally valid means of assessment [4,17-21]. Nevertheless, knowledge about the detailed evaluation of ePRO apps and information regarding patient acceptance, feasibility, and barriers are still limited [22], especially in relation to sociodemographic aspects, health status, and technical skills [7,23-26]. It also remains unclear how ePRO questionnaires are accepted and evaluated in breast cancer patients, in whom high patient satisfaction with use and usability are important implementation prerequisites for capturing real-world evidence in routine clinical care.

### Aims and Objectives

The aim of this study was to analyze the acceptance and evaluation of a tablet-based ePRO app for breast cancer patients. More specifically, we investigated how suitable patients maintain an app that is used to collect HRQoL data, whether they find it diffuse or difficult to use, and how to evaluate individual aspects of the questionnaire. To determine whether the HRQoL survey app can be used in all breast cancer patients, we also examined whether the app’s suitability, effort, and difficulty ratings were dependent on HRQoL and sociodemographic factors (age, educational status, and computer skills).

## Methods

### Study Design and Sample

The methodology has already been described in detail elsewhere [19,21]. Here, it was shown that HRQoL can be validly assessed by the related tool, since no significant differences in response behavior between pPROs and ePROs were found with regard to reliability in both the European Organization for the Research and Treatment of Cancer core quality of life questionnaire (EORTC QLQ-C30) and the Functional Assessment of Cancer Therapy-Breast Cancer (FACT-B) questionnaires. In this study, the feasibility and acceptability will be evaluated. For digital assessment, we used a web-based solution PiiA (patient interactively informs doctor), allowing patients to answer the HRQoL assessment on a tablet after receiving anonymized user credentials [19,21]. Patients were recruited as a part of the ePROCOM (electronic Patient-Reported Outcomes and Compliance Analysis) and the PEPPER (Patient Engagement Breast Cancer) study. While the ePROCOM study aims to evaluate general patient acceptance and practicability of a web-based app for a PRO questionnaire for patients with adjuvant or metastatic breast cancer, the PEPPER study aims to evaluate the impact of web-based PROs and pPROs for health care services. The inclusion criteria were female gender, full legal age, proven diagnosis of breast cancer in an adjuvant or metastatic setting, sufficient language skills in German, and signed declaration of consent. The exclusion criterion was participation in other studies to minimize the burden of questionnaires. Patients were asked to complete the questionnaire during an outpatient visit at the hospital under the supervision of an attending physician. From July 2015 to May 2016, questionnaires were completed by a total of 106 female adjuvant and metastatic breast cancer patients treated consecutively at the Department of Women’s Health in Tubingen, Germany, and the National Center for Tumor Diseases (NCT) in Heidelberg, Germany. The study was designed as a 2-center prospective trial (Tübingen and Heidelberg). Ethical approval was granted by the Ethics Committee of the University of Heidelberg (S-569/2015) and the Ethics Committee of the University of Tübingen (project number 089/2015B01).

### Questionnaires

All patients were required to complete both the ePRO and pPRO versions of the EORTC QLQ-C30 and FACT-B HRQoL questionnaires. The reliability of a tablet-based ePRO app of both questionnaires has already been analyzed (the results of the reliability analysis have been published previously [19,21]). Furthermore, patients were questioned about pre-existing technical skills, their willingness to use ePROs, potential barriers in relation to their health status, and socioeconomic variables [7,26]. Thereafter, patients were asked to evaluate the app and its handling, which is reported in the current paper. The questionnaires for measuring socioeconomic status and evaluating the app were developed by our own research group. Regarding the evaluation of the app, patients assessed the ePRO questionnaires in terms of suitability, effort, and difficulty compared with the pPRO questionnaires. They also mentioned whether the introduction of ePROs was thought to have a positive impact on the quality of health care and how they considered the app in terms of usability, graphic design, and applicability. Patients were informed about the aims of the study and were asked for their consent ex ante.

### Statistical Analyses

All statistical analyses were conducted using IBM SPSS Statistics (Version 24). First, a frequency analysis was performed to determine the descriptive sociodemographic characteristics of the patients. Subsequently, the mean values and dispersion parameters of the variable age were calculated, and then, the frequencies of the individual dimensions of the variable educational attainment were determined. Thereafter, HRQoL from the EORTC QLQ-C30 questionnaire [21] and computer skills of the patients were assessed before the evaluation sheets were analyzed descriptively. Thereby, stratification was done by treatment setting, using the Mann-Whitney *U* test to test the significance of the identified frequency differences. Subsequently, multivariable regression analyses were conducted on the 3 aforementioned target outcomes. The aim of regression analysis was to determine whether the regression models showed statistically significant relationships between the evaluation dimensions of suitability, effort, and difficulty and between socioeconomic variables (age and educational attainment), computer skills, the treatment regimen (metastatic vs adjuvant), and HRQoL. For each level of ordinal variables, dummy variables were created in multivariable regression analyses. In all analyses, *P* values <.05 (2-tailed) were considered indicative of statistically significant differences (α=.05).

## Results

### Sociodemographic Variables

Table 1 shows the sociodemographic characteristics of the study group stratified by therapy setting with 76 (72%) patients in adjuvant therapy and 30 (28%) with metastatic disease, as well as their HRQoL, their self-related computer skills, and their computer use experience in years. There were no significant differences between adjuvant and metastatic patients. The mean age was 49.4 years in the adjuvant group and 53.9 years in the metastatic group. Nearly half of the patients (adjuvant patients: 38/76, 50%; metastatic patients: 14/30, 47%) had a higher level of education (advanced technical graduation or high school diploma). The mean HRQoL score was approximately 60 points in both groups (where 0 represents the worst value and 100 the highest value). Among all patients, more than three-quarters rated their computer skills as advanced or professional (adjuvant patients: 54/76, 71%; metastatic patients: 20/30, 67%), while the mean time of computer use was more than 10 years in both groups.

**Table 1:** Sociodemographic characteristics of the patients.

Sociodemographic variables			Adjuvant therapy group (n=76, 72%)		Metastatic situation group (n=30, 28%)		*P* value
**Age (years)**							.18
	Mean value (SD)	49.39 (10.28)		53.93 (13.94)			
	Median value (minimum-maximum)	50.0 (27-73)		52.0 (33-84)			
**Level of education, n (%)**							.37
	No qualification	0 (0)		1 (3)			
	Main/secondary school graduation	30 (39)		11 (37)			
	Advanced technical graduation	11 (14)		8 (27)			
	High school diploma (“Abitur”)	27 (36)		6 (20)			
	Not specified	8 (11)		4 (13)			
**HRQoL^a^ (overall HRQoL from EORTC QLQ-C30^b^)**							.16
	Mean value (SD)	63.51 (23.26)		57.77 (19.27)			
	Median value (minimum-maximum)	67 (17-100)		62.5 (17-100)			
**Computer skills (self-perception by the patients)**							.61
	Mean value (SD)	2.82 (0.64)		2.69 (0.62)			
**Computer skills level, n (%)**							
	Beginner (1)	2 (3)		2 (7)			
	Basic (2)	16 (21)		4 (13)			
	Advanced (3)	47 (62)		20 (67)			
	Professional (4)	7 (9)		0 (0)			
	Not specified	4 (5)		4 (13)			
**Computer use (years)**							.18
	Mean value (SD)	17.50 (7.09)		14.22 (9.59)			
	Median value (minimum-maximum)	18 (0-35)		15 (0-35)			

^a^HRQoL: health-related quality of life.

^b^EORTC QLQ-C30: European Organization for the Research and Treatment of Cancer core quality of life questionnaire.

### Evaluation of Suitability, Effort, and Difficulty: Comparison Between ePRO and pPRO Surveys

**Figure 1:**
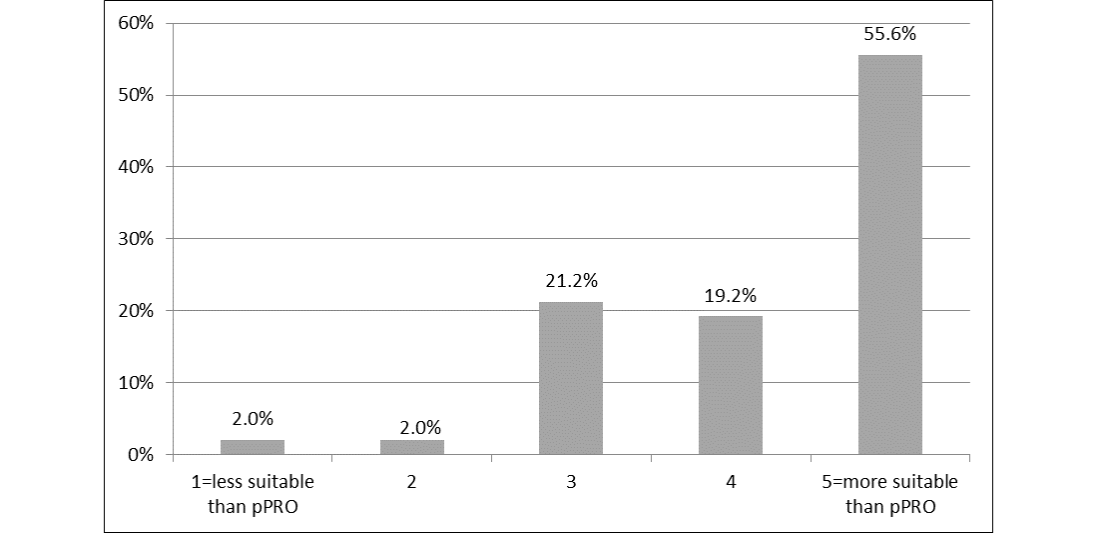
Suitability of the electronic patient-reported outcome survey in relation to the paper-based patient-reported outcome (pPRO) survey.

Figure 1 shows how the patients evaluated the app in comparison to the paper version. We examined how the platform was rated in terms of suitability compared to the pPRO survey on a 5-point Likert scale. A rating of 3 was considered as comparable suitability between the 2 assessment strategies, whereas a rating of 1 or 2 was regarded as low suitability and a rating of 4 or 5 was regarded as high suitability. Three-quarters of the patients (80/106, 75.5%) reported that the ePRO survey on the EORTC QLQ-C30 and the FACT-B questionnaire was more appropriate than the pPRO survey.

**Figure 2:**
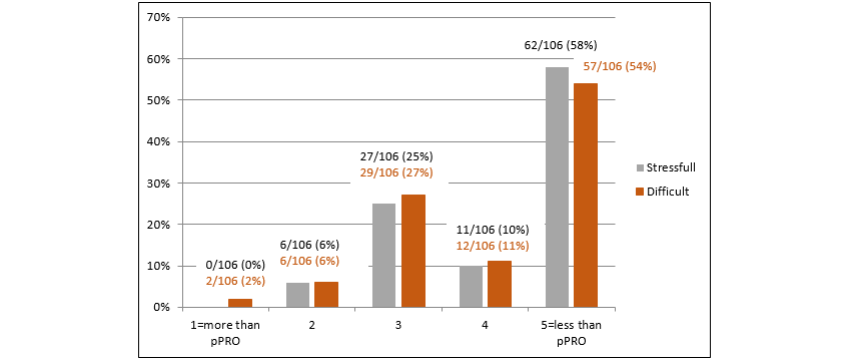
Effort and difficulty of the electronic patient-reported outcome survey in relation to the paper-based patient-reported outcome (pPRO) survey.

Similar results were obtained in the dimensions effort and difficulty. One-quarter of the patients stated that completing the ePRO sheets was as stressful (27/106, 25.5%) and as difficult (29/106, 27.4%) as completing the HRQoL questionnaires on paper, whereas 68.9% (73/106) of the patients rated the ePRO survey as less stressful and 65.1% (69/106) as less difficult than the pPRO survey. The proportion of patients who rated the ePRO survey worse than the pPRO survey was negligible (Figure 2).

Overall, 82.1% (87/106) of patients said that the introduction of the ePRO survey improved health care in hospitals, 16.0% (17/106) of patients said that the introduction of the ePRO survey was associated with deterioration in health care, and 1.9% (2/106) of patients said that the introduction of the ePRO survey had no impact on health care in hospitals.

### Influential Factors in ePRO Evaluation

For the suitability, effort, and difficulty of the ePRO survey, we examined whether the respective evaluation was influenced by socioeconomic factors, HRQoL, therapeutic setting, self-assessed computer knowledge, or experience in using computer technology. In the suitability dimension, no statistically significant correlations were found in the multivariable regression analysis. Statistically significant regression correlations were found between evaluation in the effort and difficulty dimensions and the educational level, as well as the time span of computer technology use. With higher levels of education and increasing time of using computer technology, completing the ePRO survey was more often reported as requiring less effort and being less difficult than completing paper questionnaires. By contrast, age, HRQoL, therapy setting (metastasized vs adjuvant therapy), and computer skills did not influence the response behavior in the evaluation. A total of 10.5% of the assessments could be attributed to the level of education in the effort dimension assessment, and a total of 14.5% in the dimension of difficulty, while time span of computer use only influenced the evaluation with 0.2% in both dimensions (Table 2).

**Table 2:** Multivariable regression analyses on suitability, effort, and difficulty of the electronic patient-reported outcome assessment app.

Variable		*R*		*R* ^2^	*P* value		95% CI
**Dependent variable: Suitability of the ePRO^a^ assessment**							
	Therapy setting	0.152	0.0231		.55	−0.356 to 0.659	
	Age	0.007	<0.0001		.51	−0.014 to 0.029	
	Education	−0.132	0.0174		.24	−0.353 to 0.090	
	Time of computer use	0.034	0.0012		.06	−0.001 to 0.069	
	Computer skills	−0.098	0.0096		.64	−0.519 to 0.323	
	HRQoL^b^	0.005	<0.0001		.97	−0.010 to 0.010	
**Dependent variable: Effort of the ePRO assessment app**							
	Therapy setting	0.136	0.0185		.58	−0.353 to 0.624	
	Age	0.014	0.0002		.16	−0.006 to 0.034	
	Education	−0.324	0.1050		.003^c^	−0.536 to −0.113	
	Time of computer use	0.043	0.0018		.01^c^	0.009 to 0.076	
	Computer skills	0.004	<0.0001		.98	−0.396 to 0.404	
	HRQoL	0.008	<0.0001		.12	−0.002 to 0.017	
**Dependent variable: Difficulty of the ePRO assessment app**							
	Therapy setting	0.071	0.0050		.80	−0.492 to 0.633	
	Age	0.006	<0.0001		.62	−0.017 to 0.028	
	Education	−0.381	0.1451		.003^c^	−0.626 to −0.136	
	Time of computer use	0.040	0.0016		.04^c^	0.002 to 0.079	
	Computer skills	0.093	0.0086		.69	−0.365 to 0.551	
	HRQoL	0.004	<0.0001		.50	−0.007 to 0.015	

^a^ePRO: electronic patient-reported outcome.

^b^HRQoL: health-related quality of life.

^c^Statistically significant difference.

### Evaluation of the App’s Usability

**Figure 3:**
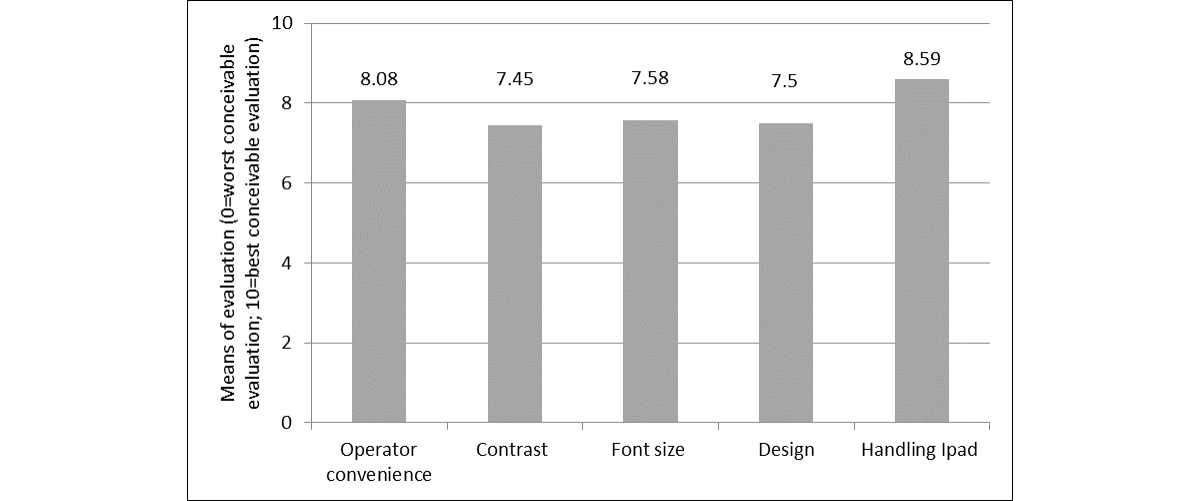
Application usability in different aspects of user experience.

Figure 3 shows the mean values of the usability evaluation. All 5 dimensions had high to very high ratings. The dimensions operator convenience, contrast, font size, and design were scored between 7.4 and 8.1, while handling was scored at 8.6 on average.

## Discussion

### Principal Findings

The results indicated that ePRO surveys are also applicable for measuring HRQoL in breast cancer patients with metastatic disease or under adjuvant therapy. The app was rated as more suitable, as requiring an equal or lesser degree of effort, and as being equally or less difficult than the pPRO survey. The evaluation of usability and applicability showed high to very high ratings. Thus, it could be shown that the ePRO survey can be used even in patients with a high burden of disease as well as in older patients, as HRQoL and age did not affect the evaluation. This is a significant finding, which extends the previous state of research, as it previously appeared unclear whether there were barriers to using ePROs in elderly and metastatic patients [7]. The influence of educational level on the evaluation was significant but rather small.

### Comparison With Prior Work

Although ePRO apps are being adopted more frequently, paper-based surveys of PROs still predominate in clinical research because reliable electronically validated questionnaires are lacking. This is why knowledge about the detailed evaluation of ePRO apps and information regarding patient acceptance, feasibility, and barriers are still limited [22], although the potential of ePROs is high and the experience is very satisfactory so far [27]. The results of this study basically confirm the results of few existing studies [4,24,28,29]. Wintner et al likewise showed that cancer patients preferred ePRO questionnaires over pPRO questionnaires [28]. A high rating could also be found for electronic psycho-oncological screening instruments in breast cancer patients; here, the acceptance was greater than that of the paper-pencil screening [24]. However, only results from very small patient populations are available for breast cancer patients with metastatic disease or under adjuvant therapy. Both a Japanese and a German research group were able to demonstrate positive effects in this area. However, the number of patients included was less than 20 [4,29]. The current results therefore represent a unique characteristic, as we were able to demonstrate for the first time that ePRO surveys are also well received and better evaluated than paper-based surveys by patients with a high burden of disease in a larger collective. Other studies have not yet focused on the factors that influence app evaluation (and thus the response behavior of patients [22]). The fact that the level of education and the time span of using computer technology influence the evaluation of ePROs confirms the findings of our team, as the willingness to use such an app is also influenced by socioeconomic factors and computer skills [7,26].

### Limitations

Despite positive results, some limitations of the study design and methodological implementation should be mentioned, which could possibly reduce the representativeness of the data. Patients were required to complete questionnaires during an outpatient hospital visit. The phenomenon of socially desirable response behavior might have influenced the evaluation results, such that the app might have been rated differently by patients in their home environment. It also needs to be noted that there might have been selection bias, as we did not examine whether the HRQoL was lower and the psychological distress was higher in those patients who could not be motivated to participate in the study. Only patients who were already technically inclined might have been willing to participate. Therefore, it remains unclear how acceptance differs from that in patients who only display a low level of use willingness [7,26].

### Conclusions

Although digital assessment of HRQoL is constantly being adopted in clinical research and clinical routine, knowledge about the evaluation and acceptance of ePRO apps in breast cancer patients with a high burden of disease is insufficient. The results of this study indicate that breast cancer patients with metastatic disease and those under adjuvant therapy prefer ePRO surveys to pPRO surveys. However, the evaluation of ePROs depends on the level of education and the patient’s computer skills and experience. Here, studies with larger collectives are needed to develop low-threshold offers that make ePRO surveys usable for all patient groups in both clinical and home settings and to better understand the needs of patients with a higher disease burden.

## Abbreviations

EORTC QLQ-C30: European Organization for the Research and Treatment of Cancer core quality of life questionnaire
ePRO: electronic patient-reported outcome
ePROCOM: electronic Patient-Reported Outcomes and Compliance Analysis
FACT-B: Functional Assessment of Cancer Therapy-Breast Cancer
HRQoL: health-related quality of life
pPRO: paper-based patient-reported outcome
PRO: patient-reported outcome
